# Supplementary material for: Exploring the Quality and Application Potential of the Remaining Tea Stems after the Postharvest Tea Leaves: The Example of Lu’an Guapian Tea (Camellia sinensis L.)
Source: Foods. 2022 Aug 6;11(15):2357. doi: 10.3390/foods11152357 (PMC9368606; doi:10.3390/foods11152357)
Supplement: Supplementary file 1 [file foods-11-02357-s001.zip › foods-1830827-supplementary.pdf]

Table S1 Parameters of the calibration curves for the components and their limits of detection (LOD) and limits of quantification (LOQ)

Compound	Quantification curves	R^2	LOD μ g/mL	LOQ μ g/mL
Total tea polyphenol	y=0.0124x	0.9995	10.00	50.00
Total free amino acids	y=3.0570x-0.4148	0.9991	200.00	600.00
Caffeine	y=27.454x-17.722	0.9999	50.00	150.00
Total soluble sugar	y=180.35x-4.1682	0.9999	20.00	160.00
EGC	y=2.0616x-15.127	0.9999	100.00	300.00
C	y=6.5995x-23.179	0.9991	50.00	150.00
EC	y=7.3643x-17.64	0.9998	50.00	150.00
EGCG	y=12.675x-50.625	0.9999	100.00	400.00
ECG	y=13.901x-140.86	0.9999	50.00	180.00
L-theanine	y = 4472.3x -7.1252	0.9998	50.00	500.00

Note: The absorbance (A) or peak area is the vertical coordinate and the concentration of the substance is the horizontal coordinate.

Table S2 Parameters of the calibration curves for the free amino acids and their limits of detection (LOD) and limits of quantification (LOQ)

**Compound**	**Molecular weight**	**Quantification curves**	**R^2**	**LOD μ mol/mL**	**LOQ μ mol/mL**
Aspartic	133	y = 745x - 8.25	0.9978	0.02	0.20
Serine	105	y = 817.58x + 0.0583	0.9978	0.02	0.20
Glutamic	147	y = 733.67x - 0.7667	0.9961	0.02	.020
Glycine	75	y = 814.75x + 0.7417	0.9948	0.02	0.20
Histidine	155	y = 1682.3x + 9.6	0.9952	0.02	0.20
Arginine	174	y = 967x + 0.6333	0.9980	0.02	0.20
Threonine	119	y = 1144.6x - 13.775	0.9922	0.02	0.20
Proline	115	y = 979.25x - 5.4917	0.9962	0.02	0.20
Cysteine	121	y = 1043.4x + 2.175	0.9994	0.02	0.20
Tyrosine	181	y = 949.58x - 4.2417	0.9973	0.02	0.20
Valine	117	y = 1075.1x - 3.175	0.9990	0.02	0.20
Lysine	146	y = 1049.3x - 7.225	0.9966	0.02	0.20
Isoleucine	131	y = 1110.8x - 12.833	0.9918	0.02	0.20
Leucine	131	y = 1877.3x - 14.767	0.9935	0.02	0.20
Phenylalanine	165	y = 941.64x - 2.3505	0.9992	0.02	0.20

Note: The peak area is the vertical coordinate and the concentration of the substance is the horizontal coordinate.

Table S3 Parameters of the calibration curves for the monosaccharides and disaccharide and their limits of detection (LOD) and limits of quantification (LOQ)

Compound	Class	Quantification curves	R^2	LOD μ g/mL	LOQ μ g/mL
Lactose	disaccharide	y = 0.024821 x - 7.929386E-006	0.9981	0.003	0.300
Sucrose	disaccharide	y = 0.032313 x - 0.014041	0.9952	0.300	40.00
Trehalose	disaccharide	y = 0.138757 x - 2.791236E-004	0.9965	0.003	0.300
Maltose	disaccharide	y = 0.022863 x + 5.287298E-006	0.9962	0.003	0.300
D-Arabinose	monosaccharide	y = 0.111964 x - 2.482961E-004	0.9991	0.003	6.000
D-Fructose	monosaccharide	y = 0.007089 x - 6.196312E-005	0.9993	0.006	6.000
L-Fucose	monosaccharide	y = 0.085968 x - 1.988209E-004	0.9927	0.003	0.300
D-Galactose	monosaccharide	y = 0.001116 x - 3.672196E-006	0.9956	0.003	0.300
Glucose	monosaccharide	y = 0.109420 x - 7.648392E-004	0.9922	0.006	6.000
Inositol	monosaccharide	y = 0.045961 x - 9.790822E-005	0.9965	0.003	1.500
L-Rhamnose	monosaccharide	y = 0.012280 x + 5.712896E-005	0.9957	0.003	0.300
D-Sorbitol	monosaccharide	y = 0.160173 x - 7.038672E-004	0.9971	0.006	6.000
Xylitol	monosaccharide	y = 0.069807 x - 9.152151E-005	0.9950	0.003	0.300

Note: The concentration ratio of the external and internal standards is used as the horizontal coordinate and the ratio of the peak area of the external and internal standards as the vertical coordinate.
